# Fungal Carboxymethyl Chitosan-Impregnated Bacterial Cellulose Hydrogel as Wound-Dressing Agent

**DOI:** 10.3390/gels9030184

**Published:** 2023-02-27

**Authors:** Maduru Suneetha, So-Yeon Won, Sun Mi Zo, Sung Soo Han

**Affiliations:** 1 School of Chemical Engineering, Yeungnam University, 280 Daehak-Ro, Gyeongsan 38541, Republic of Korea; 2 Research Institute of Cell Culture, Yeungnam University, 280 Daehak-Ro, Gyeongsan 38541, Republic of Korea

**Keywords:** bacterial cellulose, fungal carboxymethyl chitosan, hydrogel, antibacterial, wound dressing

## Abstract

Bacterial cellulose (BC) produced by *Gluconoacetobacter hansenii* is a suitable polymeric fiber network for wound-dressing purposes, but its lack of antibacterial properties limits it from healing bacterial wounds. We developed hydrogels by impregnating fungal-derived carboxymethyl chitosan to BC fiber networks using a simple solution immersion method. The CMCS–BC hydrogels were characterized using various characterization techniques such as XRD, FTIR, water contact angle measurements, TGA, and SEM to know the physiochemical properties. The results show that the impregnation of CMCS into BC fiber networks greatly influences BC’s improving hydrophilic nature, which is crucial for wound healing applications. Furthermore, the CMCS–BC hydrogels were studied for biocompatibility analysis with skin fibroblast cells. The results revealed that by increasing the CMCS content in the BC, biocompatibility, cell attachment, and spreading capacity also increase. The antibacterial activity of CMCS–BC hydrogels is shown using the CFU method against *Escherichia coli* (*E. coli*) and *Staphylococcus aureus* (*S. aureus*). As a result, the CMCS–BC hydrogels exhibit more suitable antibacterial properties than those without BC due to the CMCS having amino groups that enhance antibacterial properties. Therefore, CMCS–BC hydrogels can be considered suitable for antibacterial wound dressing applications.

## 1. Introduction

Cellulose (CS) is a common water-insoluble polysaccharide found in nature. Although cellulose is most commonly sourced from plants, it is also produced by various microorganisms such as fungi, algae, and bacteria [1]. The biosynthesized bacterial cellulose (BC), similar to plant cellulose structure, is a nearly pure form of cellulose produced by strains of the Gluconacetobacter hansenii PJK is a Gram-negative bacterium using glucose as a substrate for producing bacteria [2]. BC has many properties compared to plant cellulose, such as high water-holding ability, nontoxic crystallinity, an ultrafine fiber network, and the ability to be molded into three-dimensional (3D) structures during synthesis [3]. Recently, there is also an increasing interest in developing a wide range of biomedical applications, such as artificial blood vessels, scaffolds in tissue engineering, burns, dental implants, and wound dressing for wounds [4,5,6,7]. It not only has biomedical applications but also some other applications such as to food, paper, acoustic, filter membrane, pharmaceutical industries, and environmental applications [8]. BC has been extensively researched, particularly for use in wound-dressing materials [9]. These wound-dressing materials could provide the following advantages: wound infection prevention, high gas exchange, cell respiration permission, replicating the natural extracellular matrix structure (ECM), good wound exudate absorption capacity, quick and painless removal, accelerating the healing process, and being nontoxic and nonallergenic [9,10]. Although BC has excellent properties for wound dressing, it lacks antibacterial properties, which is crucial for infected wounds to kill bacteria during wound healing.

Naturally sourced polymers have been used for biomedical applications [11,12,13]. Among these, chitosan (CS) is a polysaccharide with abundant amino functionality derived from the deacetylation of chitin sourced from natural marine crab shells. It is widely used in the application of biomedical due to its biodegradability, biocompatibility, and nontoxicity [14]. A few reports have attempted to prepare BC-based composites with the combination of CS for wound dressing application. Although CS has the potential for wound dressing properties, it has drawbacks for preparing composites because of is insolubility in water. However, it can be modified by incorporating carboxylic functionality, glycols, and quaternized ammonium salts to improve the antibacterial properties in wound-dressing applications [15]. Among modified CS, carboxymethyl chitosan (CMCS) has been used as a potential wound dressing agent due to its excellent moisture absorbance and retention properties, biocompatibility, ease of interaction with normal cells, and excellent antibacterial activity [16]. Recently, fungal-derived CS has attracted various biomedical applications. The combination of fungal mushroom-derived CMCS has been employed in the preparation of films, nanocomposites, and hydrogels for wound-dressing applications [17,18,19,20]. Fungal-derived CMCS is more advantageous than animal-sourced CS because it has a nonallergic response; exhibits nontoxic, biodegradable, and non-ecotoxic activities; and has antibacterial properties. 

Although various forms of BC-based hydrogels have been used in wound dressing products, we herein developed a fungal-derived CMCS-impregnated BC (CMCS–BC) hydrogel, developed based on the excellent properties of CMCS sourced from fungal mushrooms. In this study, BC was produced from a bacterium called Gluconacetobacter hansenii PJK. In this study, CMCS–BC hydrogels were developed by immersing BC in CMCS solution followed by lyophilization. The lyophilized CMCS–BC hydrogels were studied for their physicochemical properties, such as surface wettability and swelling properties. Furthermore, we studied the CMCS–BC hydrogels’ biological properties, such as cell viability and antibacterial properties. Overall, the amount of CMCS content influenced the physicochemical and biological performance of the CMCS–BC hydrogels for wound-dressing applications.

## 2. Results and Discussion

### 2.1. Characterization of CMCS–BC Hydrogels

**Scheme 1:**
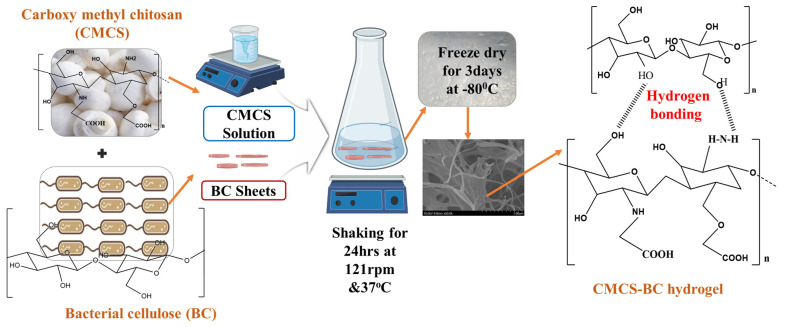
Schematic representation of CMCS–BC composite hydrogel and its hydrogen bonding interactions.

Scheme 1 represents the preparation of CMCS–BC composite hydrogel and its possible hydrogen bonding interactions. The CMCS–BC hydrogel was prepared by simply immersing BC in CMCS solution under shaking conditions. Owing to the hydrophilic functional groups such as carboxylic, amino, and hydroxyl groups on CMCS, it can easily interact with cellulose hydroxyl groups via the formation of H-bonding interactions, thereby stabilizing the CMCS–BC hydrogel. The formation of CMCS–BC hydrogel was characterized using various characterization techniques as follows.

**Figure 1:**
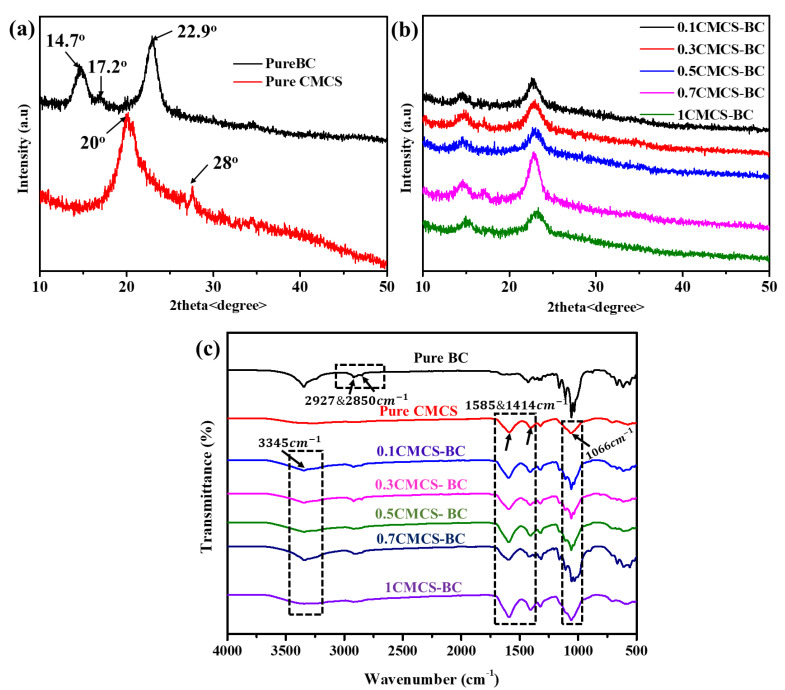
XRD patterns of pure BC and CMCS (**a**), CMCS-BC hydrogels (**b**), and (**c**) FTIR spectra of CMCS-BC hydrogels.

The XRD patterns of pure BC and CMCS–BC hydrogels are presented in Figure 1a,b. Pure BC shows crystalline peaks at 14.7°, 17.2°, and a weak shoulder peak 22.9°, which are assigned to triclinic indexation with (100), (010), and (110) planes, respectively. The intensity of the labeled peak at 2θ = 14.70 and 2θ = 17.20 and 22.90 indicates the predominant of typical crystalline and amorphous cellulose-I produced by G. hansenii. These results were in agreement with previous studies [21]. The pure CMCS shows an intense peak at 20° and 28°, which indicates a semi-crystalline structure [17]. The combination of CMCS and BC hydrogels retained the original XRD patterns of pure BC. The impregnation of CMCS into BC did not influence the structure of BC. However, the CMCS peaks disappeared in XRD patterns due to H-bonding interactions between CMCS and BC.

The FTIR spectrum of pure BC, pure CMCS, and CMCS–BC hydrogels are presented in Figure 1c. The spectrum of pure BC exhibited absorption bands at 3345 cm^−1^ for OH stretching vibration and 2940 and 2893 cm^−1^ for aliphatic C–H stretching vibration, respectively, and 1040 cm^−1^ for C–O–C stretching vibration. [1]. The spectra of pure CMCS observed peaks at 3343 cm^−1^ for stretching vibrations of the -NH groups overlapped –OH groups. At 1585 and 1414 cm^−1^, other peaks for symmetric and asymmetric stretching vibration of –COO^-^ groups. The C–O groups were observed at 1063 cm^−1^ [18]. The peaks belonging to BC and CMCS were observed for CMCS–BC hydrogels, representing the successful impregnation of CMCS into BC networks via hydrogen bonding interactions. The major peaks at 3345 and 1585 cm^−1^ for CMCS were shifted to a lower frequency region in the FTIR spectra of CMCS–BC hydrogel as CMCS amount increased in BC networks, which confirms the successful formation of hydrogen bonding interactions (–NH_2_ or –COOH) with BC (–OH) [1,21].

**Figure 2:**
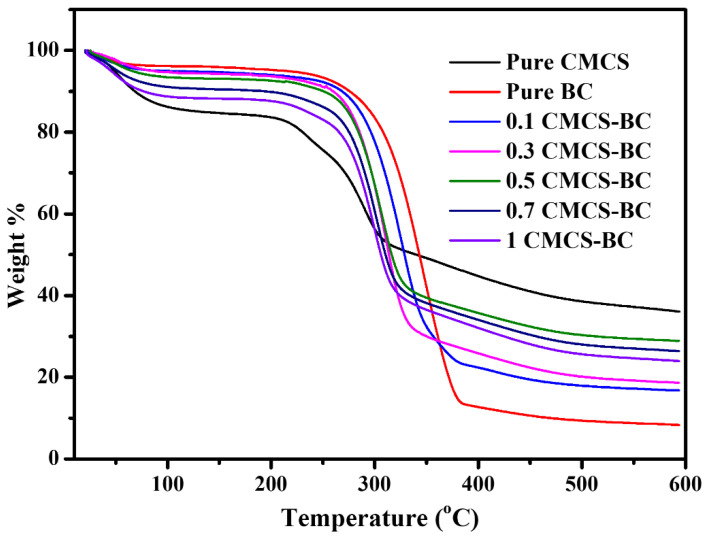
TG–DTA curves of pure BC, CMCS, and CMCS–BC hydrogels.

The thermal analysis of CMCS–BC hydrogel is an important property of application in biomedicine, which requires antiseptic treatment at high temperatures. The thermal stability and degradation characteristics of pure BC, pure CMCS, and CMCS–BC hydrogels with varying amounts of CMCS were characterized using TGA analysis. The results are presented in Figure 2. The weight loss of pure BC occurred in the TG curve at ~100 °C, which could be attributed to an evaporation of absorbed water (dehydration) molecules. The weight loss in the range between 300–400 °C due to the rapid cleavage of glycosidic bond (decomposition and depolymerization of glycosyl units) of bacterial cellulose (BC) [21]. Similarly, the initial decomposition of CMCS polysaccharide at 100 °C due to water loss. Secondly, weight loss occurred in the range of 200–320 °C due to the decomposition of the carboxylic groups and glucosamine units of the polysaccharide structure [22]. The CMCS–BC hydrogel indicates three stages of weight loss. The first stage of weight loss up to 100 °C is due to retained water dehydration. The second stage of weight loss in the range between 130–230 °C, due to the decomposition of the polymer, and the third stage of thermal decomposition of the polymer structure, might be attributed to weight loss from 250 to 400 °C [21,23]. The difference in the weight loss of the pure BC and CMCS–BC hydrogels for formulations of 0.1CMCS–BC, 0.3CMCS–BC, 0.5CMCS–BC, 0.7CMCS–BC, and 1CMCS–BC % calculated as 8.5, 10.77, 20.9, 18.5, and 16.2% respectively. Therefore, the thermal stability was improved by increasing the concentration of CMCS in the BC. 

**Figure 3:**
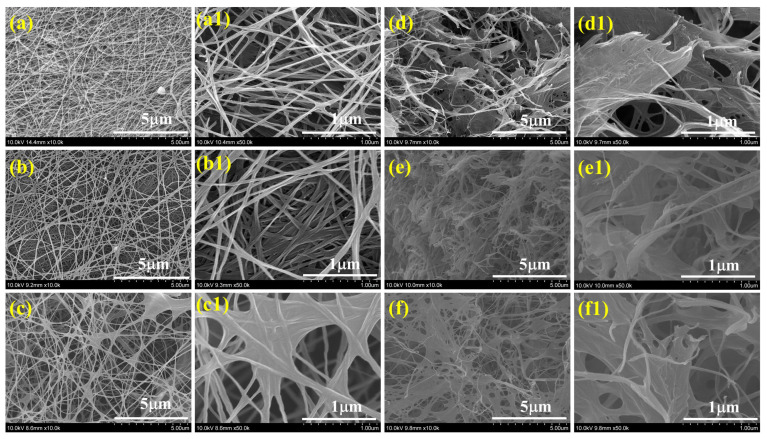
SEM images of (**a**,**a1**) pure BC, (**b**,**b1**) 0.1CMCS–BC, (**c**,**c1**) 0.3CMCS–BC, (**d**,**d1**) 0.5CMCS–BC, (**e**,**e1**) 0.7CMCS–BC, (**f**,**f1**) 1CMCS–BC hydrogels.

Figure 3 displayed the morphology of freeze-dried pure BC and CMCS–BC hydrogels were acquired using SEM. The SEM images of BC showed the nanoporous structure of a three-dimensional network with the random assembly of ribbon-shaped fibers (a and a-1) [24]. The CMCS–BC hydrogels represent the fibrous network structures, representing the CMCS polymer chains that are well-connected to BC fibers. The increasing amount of CMCS in the BC fiber networks increases the CMCS polymer chains well-connected with BC fiber due to the CMCS having a hydrophilic group and increasing hydrophilic character of cellulose fibers in the CMCS–BC hydrogels [25]. These fibrous and interconnecting porous structures allow for cell recruitment to aid in the penetration of nutrients and oxygen for wound-dressing purposes.

**Figure 4:**
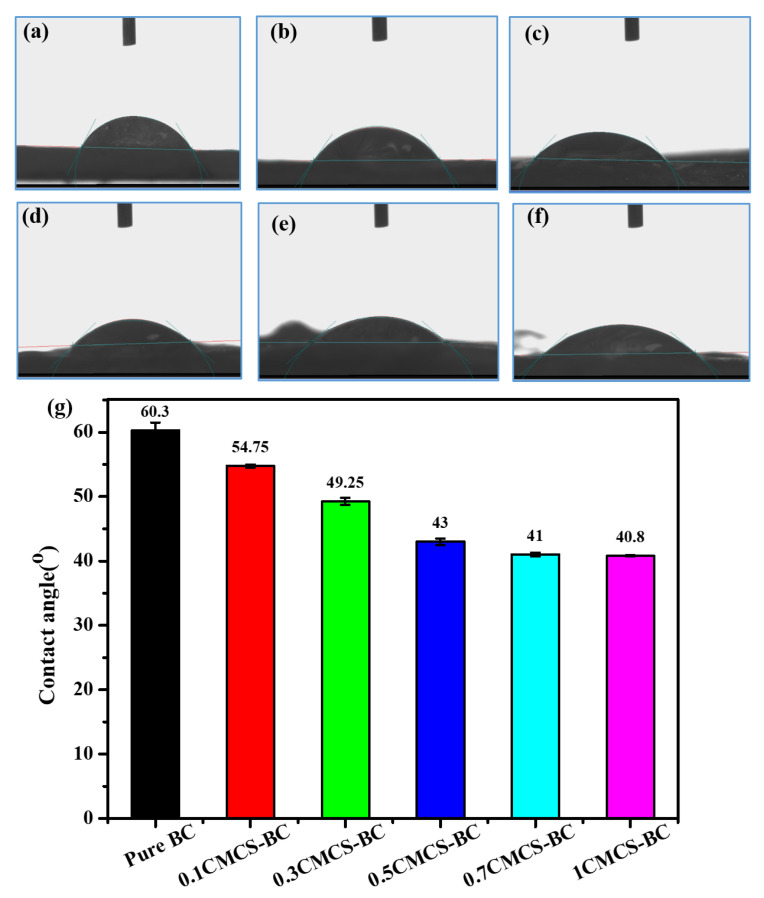
Water contact angle of (**a**) pure BC, (**b**) 0.1CMCS–BC, (**c**) 0.3CMCS–BC, (**d**) 0.5CMCS–BC, (**e**) 0.7CMCS–BC, and (**f**) 1CMCS–BC hydrogels and (**g**) their average values.

The surface wettability of wound dressing material is important for applying a wound dressing. For this, the WCA of CMCS–BC hydrogel formulations was determined to <60°, representing the water spreading over the surfaces due to the high hydrophilic characteristics of CMCS–BC wound dressing material [20]. The pure BC sheets show a WCA is about 60.3 + 1.3. Adding CMCS (0.1, 0.3, 05, 0.7, and 1%) to BC increases the WCA values 54.7 + 0.25, 49.2 + 0.5, 43 + 0.3, 41 + 0.2, 40.8 + 1.20, for 0.1CMCS–BC, 0.3CMCS–BC, 0.7CMCS–BC, and 1CMCS–BC, respectively (Figure 4). The impregnation of CMCS into BC networks improves the hydrophilicity, which is crucial for the absorption of wound extrudate. Furthermore, the CMCS–BC hydrogel can provide nutrients for improving cell adhesion and growth of cells [17]. 

### 2.2. Swelling Study

**Figure 5:**
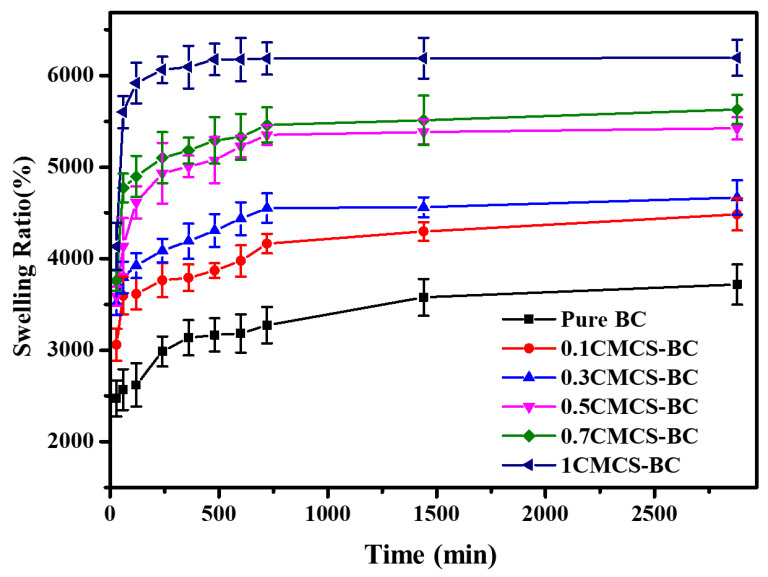
Swelling properties of CMCS–BC hydrogels.

Swelling property is also important for wound-dressing purposes. An effective wound dressing should seal the exudate and keep the wound site moist during the dressing process. A moist environment supports the penetration of active ingredients, prevents bacterial invasion, and enables painless removal from the wound surface after healing [20]. For this, the swelling behavior of the CMCS–BC hydrogel dressings was measured and shown in Figure 5. The hydrogel dressing easily absorbed the water within 2 h of incubation. The swelling of CMCS–BC hydrogel was significantly improved with the amount of CMCS due to increased hydrophilic characteristics of the BC and pore filling of water molecules [9,15]. 

### 2.3. Biocompatibility Analysis

**Figure 6:**
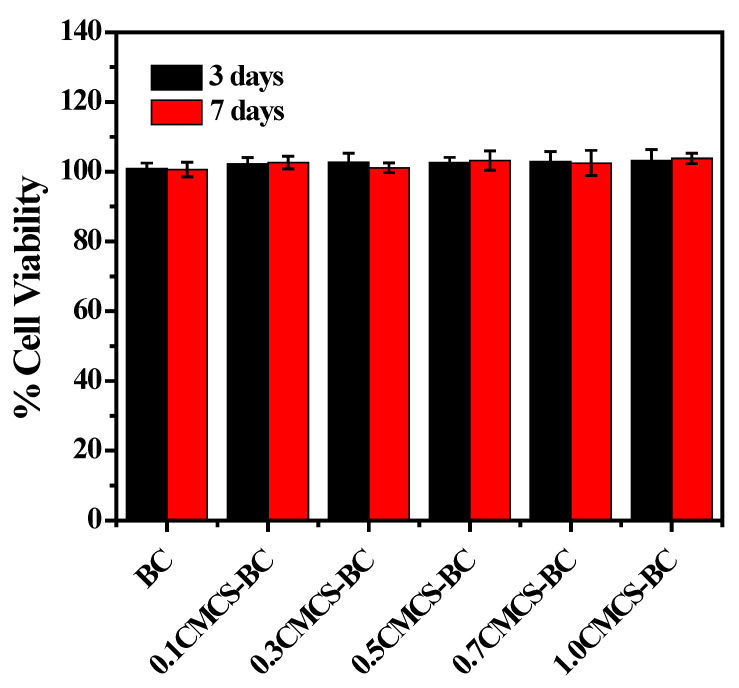
MTT assay cell proliferation of hydrogels for skin fibroblast cells.

The biocompatibility of the dressing material is critical in wound dressing application. Fibroblasts (SF) are the most common cells in skin tissue and play an important role in wound healing by producing ECM. The CMCS–BC hydrogel combination is expected to promote cell viability compared to pure BC. The proliferation of SF cells seeded on hydrogels for 1, and 3 days was assessed using an MTT test (Figure 6). All the samples do not affect the fibroblast cell viability because CMCS is a biocompatible material with amino, hydroxyl, and carboxyl groups to retain cell viability when treated with hydrogels [17,18,19,20]. 

**Figure 7:**
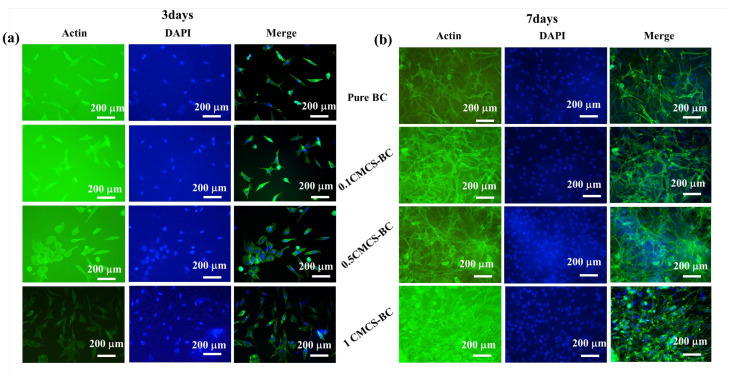
Fluorescence images of stained fibroblasts cells on pure BC and CMCS–BC hydrogels for (**a**) 3 and (**b**) 7 days under 10× magnification (scale bar 200 μm). Cell nuclei were stained with DAPI (blue), and the actin cytoskeleton was stained with Alexa flour-conjugated phalloidin (green).

The F-actin (green)/DAPI (blue) of pure BC and CMCS–BC hydrogels on SF cells displayed well-defined actin stress fibers distributed around individual nuclei of culture and areas of cell multilayering. At 3 days, the SF cells exhibited a well-spread adhesion, and some of them showed typical shapes of directional cell movement with CMCS–BC compared to pure BC (Figure 7a). At 7 days, pure BC and CMCS–BC hydrogels showed cell confluence and areas of cell multilayering (Figure 7b). The CMCS content influences the cell adhesion property and for improving cell function properties to allow the transport of oxygen and nutrients. CMCS content increases the cell growth in hydrogels compared to those without CMCS [18]. Overall, the CMCS–BC hydrogel is suitable for wound dressing applications.

### 2.4. Antibacterial Property

**Figure 8:**
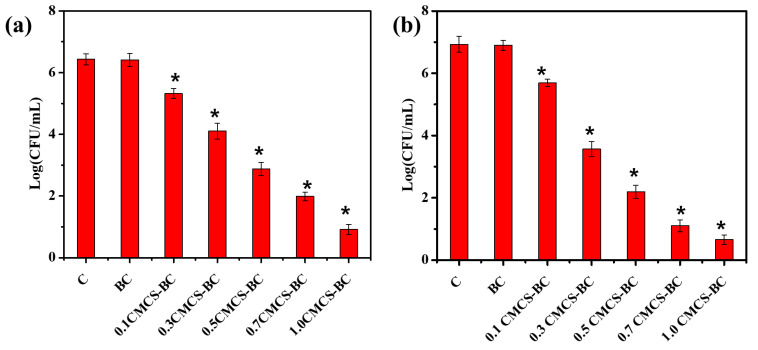
Antibacterial activity of CMCS–BC hydrogels against with (**a**) *E. coli* and (**b**) *S. aureus* bacteria (N = 3, * *p* < 0.05).

Antibacterial is an essential property in wound dressing applications. Hydrogels provide an excellent physical protective barrier to the wound site and absorb moisture content for dressing. In burn wound healing, preventing microbial infection and reducing bacterial infection is vital to improving healing rate. The antibacterial activity of CMCS–BC hydrogels is shown in Figure 8. The control without hydrogel demonstrated full growth of both bacteria. Similar to the control, BC shows bacterial growth proving BC did not have an antibacterial effect on both bacteria. According to our research, CMCS-impregnated BC hydrogel can increase its ability to kill both Gram-positive and Gram-negative bacteria. *E. coli* and *S. aureus* bacteria subjected to a higher concentration of CMCS (1%)-impregnated BC hydrogel had a greater log reduction in bacterial viability than a lower amount of CMCS (0.1%), indicating that CMCS plays a crucial role in enhanced bacterial death. The high antibacterial activity of 1CMCS–BC hydrogel formulation represents a higher amount of CMCS, which contains more amino groups that can easily kill the bacteria.

## 3. Conclusions

Bacterial cellulose (BC) and CMCS–BC hydrogels were developed in this study. The initial evaluations indicate that CMCS–BC hydrogels might be effective in wound healing. Scanning electron microscopy (SEM) showed a pure BC had a porous structure with the random assembly of interconnected cellulose fibers. The incorporation of CMCS in BC (CMCS–BC) hydrogel showed a porous structure with fibers as a result of the swelling property, which maintains a suitable moisture environment for dressing the wound. CMCS–BC hydrogels show properties of cell study (cell viability, DAPI/Actin, and antibacterial) when adding CMCS to show good biological properties. Incorporating CMCS in BC offers suitable physiological and biological activities for wound-dressing applications. 

## 4. Materials and Methods

### 4.1. Materials

Fungal mushroom-derived carboxymethyl chitosan (CMCS) deacetylation 80–98%; Viscosity 20–1000 mpas with MW = 200,000–2,000,000 Da was gifted by the Endovision Company of Daegu in South Korea. Glucose was received from SAMCHUN Company, and yeast extract and peptone were purchased from Seouchou, Seoul, Republic of Korea. Succinate was obtained from DUKSAN reagents and chemicals. Acetic acid was received from DC Chemical Co., Ltd., Seoul, Republic of Korea, Gluconacetobacter hansenii PJK (ATCC 8246 NCIB) was used in this study. 

### 4.2. Preparation of BC 

Gluconacetobacter hansenii PJK (ATCC 8246 NCIB ) was cultivated on MAE basal medium containing 10 g/L glucose, 10 g/L yeast extract, 7 g/L peptone, 1.5 mL/L acetic acid, and 0.2 g/L succinate in deionized water (DI-water) [2]. Then, the pH was adjusted to 5.0 by adding 1 M NaOH, then the medium was sterilized for 15 min at 121 °C. Hansenii PJK colonies were inoculated into 50 mL of media in a 250 mL Erlenmeyer flask, agitated at 150 rpm, and cultivated at 300 °C for 24 h. Finally inoculated bacteria was added to 1 L of media and then kept in the incubator at 37 °C for 14 days. After 14 days, the pristine BC was obtained and washed with 1 M NaOH at 121 °C for 15 min to remove bacterial cells and other substances until completely washed with DI water for neutrality.

### 4.3. Preparation of CMCS–BC

To prepare CMCS–BC hydrogels, first, CMCS solution was prepared in DI water (0.1, 0.3, 0.5, 0.7, and 1%). Then, wet BC was immersed in different % of CMCS solution for 24 h at 121 rpm at room temperature. Afterward, the samples were removed from the CMCS solutions and then freeze-dried at −80 °C for two days.

### 4.4. Characterization

The CMCS–BC hydrogels were characterized with a range 4000–500 cm^−1^ using Fourier transform infrared spectroscopy (FTIR) using the Perkin Elmer instrument, Wisconsin, MI, USA. The X-ray diffraction (XRD) patterns of the hydrogels were analyzed in the range from 2θ = 10–80° with a scanning rate of 5°/min (Xpert). The morphology of CMCS–BC hydrogels was characterized using scanning electron microscopy (SEM, Hitachi-4800). At 25 °C, the water contact angle (WCA) of the CMCS–BC hydrogels was determined using a sessile water drop method (physics instruments—OCA20). The freeze-dried hydrogels were cut into pieces and placed on a glass slide to determine the contact angle. Finally, 10 µL of DI water was placed on the CMCS–BC hydrogels, and an image was taken after 2 s. All hydrogel’s water contact angles (WCA) were tested in triplicate (n = 3). TGA analysis of CMCS–BC hydrogels was carried out using a TA instrument with an ambient temperature range of 800 °C at 10 °C/min in an inert atmosphere (nitrogen flow).

### 4.5. Swelling Property

The CMCS–BC hydrogels were cut into small pieces for swelling performances, and freeze-dried hydrogels (*W_f_*) were weighed. The cut hydrogels were immersed in DI water and incubated in an orbital shaker at 37 °C with 100 rpm. After different time intervals, the hydrogels were wiped with tissue paper to remove excess DI water and then weighed (*W_s_*). All hydrogels were tested in triplicate (n = 3); the swelling property of the hydrogels was analyzed using the following formula.

$$\mathrm{Swelling}\;\mathrm{property}\;\left(\%\right)=\frac{W_{s}-W_{f}}{W_{f}}\times 100$$

### 4.6. Biocompatibility Analyses

DMEM (Dulbecco’s modified Eagle’s medium) containing 10% FBS and 1% penicillin G-streptomycin was used to culture skin fibroblasts (SF) (ATCC). The effect of CMCS–BC hydrogels on cell proliferation was evaluated using the MTT assay and L/D assay (Life Technology, USA-live/dead assay kit—Life Technology, Carlsbad, CA, USA).

For cell proliferation, SF cells were seeded at a density of 5 × 10^4^ cells/cm^2^ onto CMCS–BC hydrogels fixed in 24-well plates and cultured for 3 and 7 days. The media was changed every alternative day. After 3 or 7 days, the media was discarded, and 200 µL of MTT dye solution (5 mg/mL PBS solution) was added to each well and incubated at 37 °C. After 4 h, the formazan crystals were created and dissolved by adding an acidic isopropanol solution and incubating for 30 min in the dark at room temperature. The resultant solution was transferred from each well to a 96-well plate, and optical density readings were recorded at 570 nm using a microplate reader (Biotek Instrument, Winooski, VT, USA).

Furthermore, to visualize the cells grown in CMCS–BC hydrogels, the cells grown on hydrogels were visualized using fluorescence microscopy. After 3 and 7 days of culturing cells on hydrogels, the cells were fixed with 4% paraformaldehyde for 10 min, and then 0.1% Triton X-100 was added to permeabilize cell membranes. Finally, the cells were stained with phalloidin for 30 min to observe the green fluorescence of cytoskeletal filamentous actin (F-actin) and then counterstained with DAPI for 10 min. Finally, cells were washed with PBS three times. The fluorescence images of cells were acquired using fluorescence microscopy. 

### 4.7. Antibacterial Properties

The antibacterial activity of CMCS–BC hydrogels was evaluated using the CFU method. In this study, Staphylococcus aureus was the Gram-positive bacteria, and Escherichia coli the Gram-negative bacteria used. A quantity of 10^6^ CFU mL^−1^ bacteria in 4 mL Luria–Bertani (LB) broth media was fixed for the antibacterial activity of CMCS–BC hydrogels. The hydrogel samples were added to bacteria and incubated for 24 h at 37 °C with a constant rotation speed (100 rpm). Then, 200 µL bacterial solution was spread uniformly on agar plates. The bacterial colonies formed on agar plates were counted after incubating agar plates at 37 °C for another 24 h. For statistical analysis, all the data (N = 3) are presented to assess the statistical differences using ANOVA variance (one-way analysis), and a *p*-value < 0.05 was considered to be significant.

## Data Availability

Not applicable.
